# Parent Education and Counseling (PairEd-C) Intervention to Improve Family-Centered Care: Protocol for a Prospective Acceptability Study Using the Theoretical Framework of Acceptability

**DOI:** 10.2196/54914

**Published:** 2024-10-04

**Authors:** Leul Deribe, Eshetu Girma, Nataliya Lindström, Abdulkadir Gidey, Solomon Teferra, Adamu Addissie

**Affiliations:** 1 School of Nursing and Midwifery College of Health Sciences Addis Ababa University Addis Ababa Ethiopia; 2 School of Public Health College of Health Sciences Addis Ababa University Addis Ababa Ethiopia; 3 Department of Applied Information Technology University of Gothenburg Göteborg Sweden; 4 School of Medicine College of Health Sciences Addis Ababa University Addis Ababa Ethiopia

**Keywords:** family-centered care, child cancer, theoretical framework of acceptability, education and counseling, acceptability, parent education, family centered, care service, theoretical framework, study protocol, family, health care, well-being, children, implementation, design intervention

## Abstract

**Background:**

Family-centered care (FCC) is an intervention approach based on a respectful relationship between family and health care providers (HCPs) to ensure the health and well-being of children and their families. Although HCPs have a better perception of FCC, the level of its implementation is low. Reasons for low implementation include limited understanding, lack of training, and lack of implementation guidelines and tools to support implementation. Thus, we developed the Parent Education and Counseling (PairEd-C) intervention to improve FCC in pediatric oncology settings and assess its acceptability.

**Objective:**

The objective of this study is to assess the prospective acceptability of the PairEd-C intervention using the theoretical framework of acceptability (TFA) in the pediatric oncology department in a tertiary hospital in Ethiopia.

**Methods:**

The study was conducted using an exploratory qualitative study design. We aimed to recruit 10 to 15 participants for the in-depth interview. The study participants were health service leaders working in child cancer, HCPs, social workers, and parents of children with cancer. The intervention was developed using the integration of the first phase of the Medical Research Council (MRC) framework for developing and testing complex interventions and the behavior change wheel (BCW) framework. The main PairEd-C intervention components align with the intervention functions of education, persuasion, training, environmental restructuring, modeling, and enablement, which were intended to improve FCC in the pediatric oncology unit by providing structured and comprehensive education and counseling of parents of children with cancer. The intervention was implemented by providing training for the health care team, facilitating discussion among HCPs and setting a shared plan, improving the commitment of the health care team, providing education for parents, improving parents’ capacity to attend the intervention sessions, arranging discussion among parents of children with cancer, and provision of education and counseling on distress. The HCPs working in the unit received training on the designed intervention. The trained educators and the health care provider delivered the intervention. Data will be analyzed using deductive thematic coding with a framework analysis technique based on the 7 TFA constructs. Atlas ti. version 9 will be used for data analysis.

**Results:**

Funding was acquired in 2017, and ethical clearance for conducting the study was obtained. We conducted the interviews with the study participants from December 2023 to January 2024. As of the acceptance of this protocol (June 2024), 12 study participants were interviewed. The data analysis process was started subsequently, and the manuscript will be completed and submitted for publication in early 2025.

**Conclusions:**

This acceptability study is expected to show that the designed intervention is acceptable to study participants, and the findings will be used to improve the intervention before progressing to the next step of our project.

**International Registered Report Identifier (IRRID):**

DERR1-10.2196/54914

## Introduction

Family-centered care (FCC) is a respectful relationship between family and health care providers (HCPs) to ensure the health and well-being of children and their families. It recognizes the abilities, customs, cultures, and knowledge that HCPs and families bring to the partnership. In addition to improving the patient’s and family’s experience with health care, it lowers stress, fosters better communication, lessens conflict, and enhances the health of children with long-term medical disorders. Compared with patients who do not receive FCC, those who receive FCC had improved psychosocial status, communication between family members and health care professionals, and understanding about childhood cancer [1]. Thus, FCC provides care to children and families in which all family members are acknowledged as care recipients and treatment is organized considering the family as a whole [2].

Although HCPs have a better perception of FCC, there are disparities in performance and perceptions of various FCC subdomains [3-9]. Different barriers that hinder the implementation of FCC related to health providers, organizations, and families have been reported. These barriers include limited understanding of FCC principles; communication difficulties; inadequate skills; inconsistent training; and lack of knowledge, skills, time, or tools to support the implementation of FCC [8,10,11]. In addition, a lack of policy and guidelines on FCC, poor infrastructure, poor-quality design, poor intervention content, and the burden on health providers from competing priorities have been reported [12,13]. A shortage of HCPs, a lack of time, and the absence of an FCC system were also identified as barriers [10].

To overcome these challenges and facilitate the intervention of FCC in the pediatric oncology unit, we designed a new intervention based on the local context. To develop this intervention, we identified evidence through a systematic review, explored the relevant and guiding theory for intervention development, conducted baseline studies using mixed methods, and held a series of complex intervention FCC design workshops. Finally, we developed an intervention called Parent Education and Counseling (PairEd-C) to improve the delivery of FCC in the pediatric oncology unit in Ethiopia.

One key aspect of intervention effectiveness is the extent to which the intervention is considered acceptable to those providing and receiving it. Therefore, it is essential to conduct an acceptability study before implementing a new intervention [14]. Sekhon et al [15] defined acceptability as “a multifaceted construct that reflects the extent to which people delivering or receiving a health care intervention consider it appropriate, based on anticipated or experienced cognitive and emotional responses to the intervention.” Assessing an intervention’s acceptability involves evaluating how well the target population will respond to it and how much of its components suit the needs of the target demographic and organizational setting [14,16,17]. Acceptability studies are important in developing and evaluating complex interventions [18,19]. They help reduce the risk of unsuccessful implementation, reduce distrust, improve adherence to the plan, and increase the likelihood of the intervention’s sustainability [14,16,20-23].

Thus, assessing the acceptability of the PairEd-C intervention is particularly important to make all necessary modifications before evaluating the intervention clinically and understanding how best it can be implemented. The aim of this study is, therefore, to explore the prospective acceptability of the PairEd-C intervention among HCPs and parents of children with cancer to optimize further development, evaluation, and, ultimately, its implementation.

## Methods

### Study Setting and Design

The study was conducted in the pediatric hemato-oncology unit in the Tikur Anbessa Specialized Hospital, Addis Ababa, Ethiopia. This unit is the country’s main referral center for child cancer treatment. It provides care delivered by pediatric hemato-oncologists, pediatric hemato-oncology fellows, pediatric residents, oncology nurses, and generic nurses. The unit also has social workers and psychologists. Each year, the unit provides care for about 600 new cases of children with cancer. Cancer treatment in this unit mainly involves chemotherapy, radiotherapy, and surgery.

### Study Design

A descriptive, exploratory qualitative study is being used to assess the prospective acceptability of the newly designed PairEd-C intervention using the theoretical framework of acceptability (TFA).

### Participant Recruitment

Since our objective is to assess acceptability from the perspectives of both parents of children with cancer and HCPs, the study participants were the parents of children with cancer and HCPs (physicians, nurses, social workers, and team leaders) working in the unit. In addition, policymakers were interviewed to get their perspectives on the intervention. Parents of children with cancer who visit the pediatric oncology unit and met our inclusion criteria were invited to participate in the in-depth interview. We anticipated recruiting a total of 5 parents of children with cancer. During the selection of parents, maximum variation was maintained by residence, education status, and type of childhood cancer. We planned to include 10 HCPs (physicians, nurses, and psychologists) in the in-depth interviews. During the selection of HCPs, responsibility in the unit, service year at pediatric oncology, working area, and level of education were considered. Saturation determined the final number of study participants.

### Theoretical Framework

**Figure 1:**
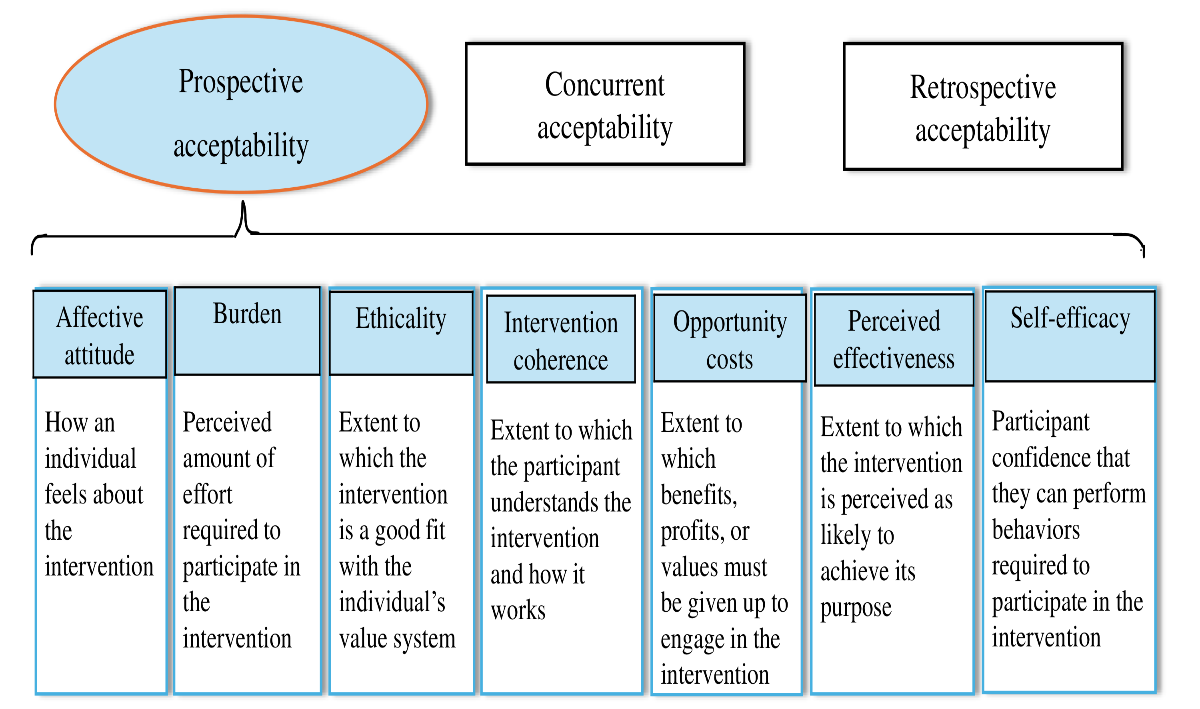
The theoretical framework of acceptability developed by Sekhon and colleagues [15].

We adapted the TFA developed by Sekhon et al [15] to guide this study [15]. The TFA is designed explicitly to assess the acceptability of health care interventions from the perspectives of people receiving and delivering interventions. It sets out a theory-informed structure based on participants’ cognitive and emotional responses [15]. It consists of 7 conceptually different constructs that capture the following essential acceptability dimensions: affective attitude, burden, ethicality, intervention coherence, opportunity costs, perceived effectiveness, and self-efficacy [15]. See Figure 1. It can be applied before, during, or after an intervention to assess prospective, concurrent, and retrospective acceptability. Since its development in 2017, it has been applied in varied contexts for these purposes [24-27]. It has also been used for structured data analysis [26,28-31], developing questionnaires [32,33], and informing an interview guide development [24,27,32-34]. The TFA can be applied using quantitative, qualitative, or mixed study approaches.

For this study, the TFA is suitable since its concepts are applicable for evaluating an intervention in individual, interpersonal, and community contexts. Similarly, the PairEd-C intervention is planned to be delivered to parents of children with cancer coming from different socioeconomic backgrounds. As a result, to evaluate it, a framework with components that are applicable to assessing how context-specific demands are met by providing a workable and culturally relevant solution must be used. Therefore, we planned to evaluate acceptability as the perception among beneficiaries and intervention implementers. This aids in determining whether the current intervention will be agreeable, palatable, or satisfactory. This will help to obtain more collective feedback about the nature of the PairEd-C intervention from different stakeholders [15,35]. In addition, assessing anticipated acceptability before participation can highlight which aspects of the intervention could be modified to increase acceptability and, thus, participation [15].

### The PairEd-C Intervention

The PairEd-C intervention was developed by integrating the Medical Research Council (MRC) [23] and behavior change wheel (BCW) [36] models. These theoretical foundations were used for similarly designed interventions in different setups. The BCW was developed from frameworks of behavior change and includes the behavior system known as COM-B at the center [36]. COM-B includes capability (C), opportunity (O), and motivation (M), which ultimately interact to produce behaviors (B) [36]. Parents’ behaviors that require modification include their intention to be involved in their child’s care, to ask and communicate with HCPs, and communicate with other parents of children with cancer. The BCW includes 9 intervention functions and 7 policy categories that support intervention design [36]. The main PairEd-C intervention components will align with the following intervention functions: education, persuasion, training, environmental restructuring, modeling, and enablement. These intervention functions are intended to improve FCC at the pediatric oncology unit through the provision of structured and comprehensive education and counseling for parents of children with cancer.

**Figure 2:**
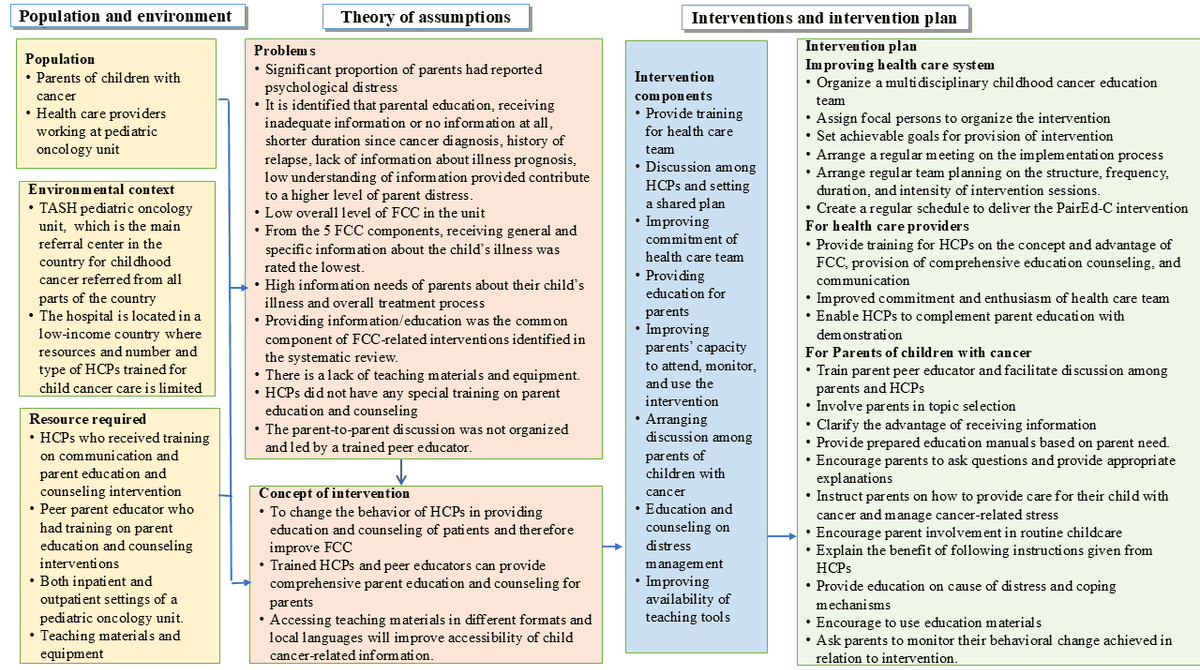
Logic model linking the context of the health care system, resources, and intervention activities [19]. FCC: family-centered care; HCP: health care provider; PairEd-C: Parent Education and Counseling; TASH: Tikur Anbessa Specialized Hospital.

**Figure 3:**
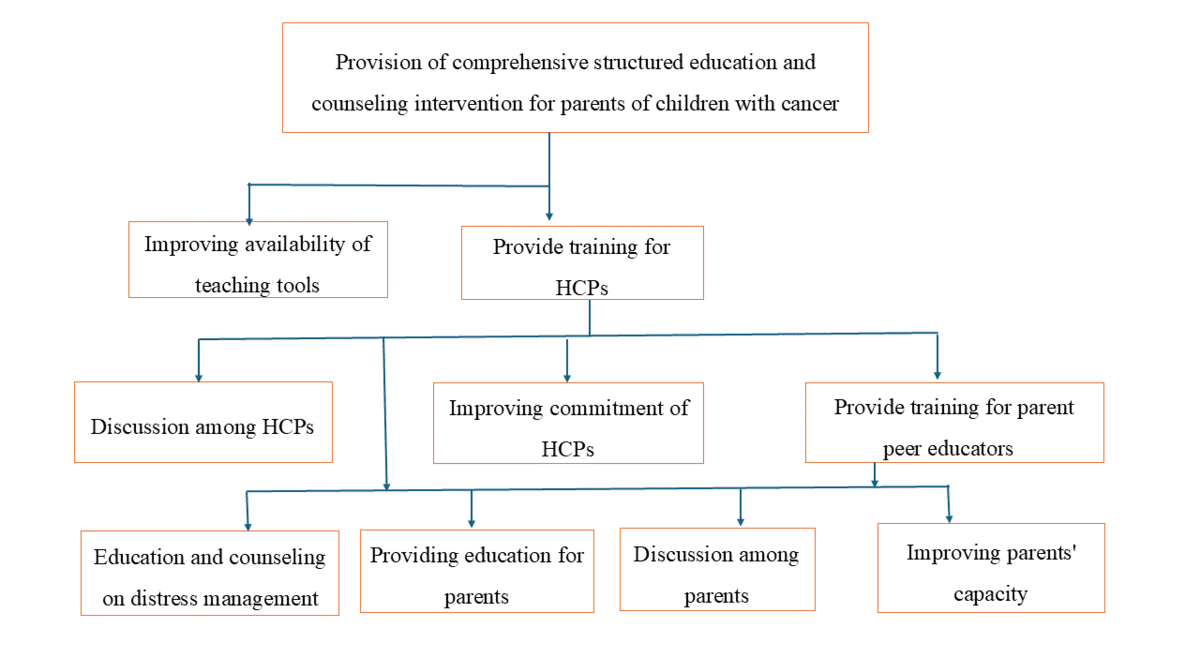
Flowchart for the implementation of intervention components to provide comprehensive structured education and counseling for parents of children with cancer. HCP: health care provider.

The main intervention components are providing training for the health care team, discussion among HCPs and setting a shared plan, improving commitment of the health care team, providing education for parents, improving parents’ capacity to attend, monitoring and using the intervention, arranging discussion among parents of children with cancer, and education and counseling on distress. The HCPs will receive training on the designed intervention. The trained HCPs will be responsible for implementing the other components, including providing training to parent-peer educators. The trained educators, with the HCP, will be involved in providing education for parents, facilitating discussion among parents, improving parenting capacity, and providing education and counseling on stress management. The detailed intervention description is presented using the Template for Intervention Description and Replication checklist. See Table 1. Figure 2 presents a logic model developed to link the health care systems context, such as the study setting, resources, intervention activities, theory and assumptions underlying the intervention, and the intervention plan, in a logical order. In addition, Figure 3 shows the implementation flow of the intervention components for the provision of comprehensive, structured education and counseling for parents of children with cancer.

**Table 1:** The Template for Intervention Description and Replication checklist [37].

Item number	Item	Item description
1	Brief name	Family-centered PairEd-C^a^ intervention for childhood cancer
2	(Why): Rationale, theory, or goal of the elements essential to the intervention	The rationale is to improve FCC^b^ at the pediatric oncology unit through the provision of structured and comprehensive parental education and counseling. Improved FCC will improve parents’ psychological health conditions. The intervention is systematically developed based on the MRC^c^ framework and the behavior change wheel model.
3	(What materials): Describe any physical or informational materials used in the intervention, including those provided to participants or used in intervention delivery or training intervention providers.	Parents will receive education and counseling on general information about cancer, cancer treatment, side effects and management of cancer treatment, providing care for a sick child at home and the hospital, and coping with a child’s diagnosis of cancer. A training manual for parents and HCPs^d^ will be provided. Parents will receive teaching aids at the end of each session.
4	(What procedures): Describe each of the procedures, activities, and/or processes used in the intervention, including any enabling or support activities.	The intervention will be implemented in 4 consecutive phases (Figure 1): phase I: preparation of setups in the pediatric oncology unit; phase II: providing training for HCPs (nurses); phase III: providing training for family peer educators; phase IV: participant selection and provision of parent education and counseling Parents will be provided with comprehensive information related to childhood cancer through a series of 12 consecutive sessions. The group discussion will follow information delivery using face-to-face counseling, videos, leaflets, and cartoon dialogs.
5	(Who provided): For each category of intervention provider (for example, psychologist, nursing assistant), describe their expertise, background, and any specific training given.	All HCPs in the pediatric oncology unit will receive training on the PairEd-C intervention for childhood cancer. Nurses with an MSc in oncology will be assigned as coordinators and lead the intervention provision. Other HCPs will help to inform and recruit parents. The trained parents will facilitate parent group discussions and help as liaisons between parents and HCPs.
6	(How): Describe the modes of delivery (such as face to face or by some other mechanism, such as internet or telephone) of the intervention and whether it was provided individually or in a group.	The intervention will be delivered using multiple approaches: Face to face: individual counseling and group discussions led by trained parents Videos, leaflets, and cartoon dialogs to provide information
7	(Where): Describe the type(s) of location(s) where the intervention occurred, including any necessary infrastructure or relevant features.	The intervention will be conducted at TASH^e^ pediatric oncology unit. Parents of children visiting inpatient and outpatient units will participate in the intervention.
8	(When and how much): Describe the number of times the intervention was delivered and over what period, including the number of sessions; their schedule; and their duration, intensity, or dose.	The PairEd-C intervention will be delivered every 2 weeks across 28 weeks. The overall intervention is classified into 12 sessions. The parents will receive the intervention when they visit the unit for regular child appointments.
9	(Tailoring): If the intervention was planned to be personalized, titrated, or adapted, describe what, why, when, and how.	The intervention schedule might be modified based on the child’s condition and treatment plan. Parents can also visit the intervention team whenever the need arises. The topic will be prioritized based on the child’s illness, treatment type, and parents’ preference. This will help to address the parents’ needs.
10	(Modifications): If the intervention was modified during the course of the study, describe the changes.	Not applicable. We are currently in the MRC framework’s design phase, and this section cannot be described until the study is complete.
11	(How well [planned]): If intervention adherence or fidelity was assessed, describe how and by whom, and if any strategies were used to maintain or improve fidelity, describe them.	Designed and assessed according to the 5 domains of the NIH^f^ Treatment Fidelity Framework
12	(How well [actual]): If intervention adherence or fidelity was assessed, describe the extent to which the intervention was delivered as planned.	Not applicable: the intervention is currently in the design phase. The pilot testing, feasibility, and intervention evaluation will be conducted in the future.

^a^PairEd-C: Parent Education and Counseling.

^b^FCC: family-centered care.

^c^MRC: Medical Research Council.

^d^HCPs: health care providers.

^e^TASH: Tikur Anbessa Specialized Hospital.

^f^NIH: National Institute of Health.

### Data Collection

Before data collection, a summarized description of the intervention prepared in the local language, Amharic, was provided to the study participants. The description of the intervention includes the major activities provided in Table 1. In addition, detailed answers were provided for any questions raised by the study participants. Data were collected in an in-depth interview using a semistructured interview guide. The interview guide was developed based on the 7 dimensions of the TFA by Sekhon et al [15] as applied to the designed intervention. See Table 2. We used recommendations from the study by Sekhon et al [38] and similar studies to develop the interview guide [24,34,39]. For instance, “burden” was explored with the question, “How easy or difficult do you think to participate in the parent education or counseling sessions?” The interview guides were pilot tested after they were independently evaluated by 2 researchers with experience in public health and complex behavior change interventions. We used the process of “back coding” to check whether the interview guide aligns with TFA constructs. A draft interview guide with opening and closing questions arranged in a random sequence and a list of the TFA structures was sent to the implementation researchers. They were asked to indicate which TFA construct each question addressed and rate how certain they were of the match on a scale from 1 to 5 (1=not at all sure; 5=sure). This process was used to assess the construct validity and whether the interview guide adequately represented the constructs in the framework [40]. All interviews were digitally recorded using a portable audio recorder and transcribed verbatim.

**Table 2:** In-depth interview guide, which was prepared using theoretical framework of acceptability (TFA) definitions to provide guidance for interviewers.

Section			Questions and prompts
**Demographic characteristics**			
	For parents	Parent age ______ Child age ______ Family sex ______F_______ Your relationship with the child ____ Your educational status ______ Residence: Urban/Rural _____ Your child’s cancer diagnosis ____ Your child’s treatment status ______ Your child’s type of treatment (chemotherapy, radiotherapy, both, off treatment, other) _______ Time since your child has been ill ___ Time since your child started treatment ______	
	For health care providers	Age ______ Sex ______ Profession ______ Service year ______ Your current position ______ Time since assigned in your current position ______	
**Introduction**			
	For parents of children with cancer	Can you talk me through the family-centered care you received at the pediatrics oncology unit? How do you explain the attention and the care you received as a parent of a child with cancer? Prompts: Information you received about your child’s illness, cancer treatment, and diagnostic procedure? During your stay in hospital or your child is treated in OPD^a^?	
	For health care providers and health leaders	Can you talk me through your care in the pediatric oncology unit? How do you explain the attention and the care you provide for parents of a child with cancer? Prompts: What information do you provide about child illness, cancer treatment, and diagnostic procedures? During their stay in hospital or child treatment in OPD?	
Affective attitude: how an individual feels about participating in the intervention			What are your overall feelings towards the planned intervention (your thoughts or feelings)? Prompts: For parents of children with new diagnoses? For parents of children with treatment follow-up? For a child on a different treatment regimen (chemotherapy/surgery)? For a parent visiting OPD? Admitted child? How comfortable did you feel receiving/providing the designed intervention?
Burden: related to self-efficacy and focuses on the perceived amount of effort required to participate in the intervention.			How much effort did it take to participate in the designed intervention? How easy or difficult do you think it is to participate in the parent education or counseling sessions? What do you think makes the intervention easy or difficult? Prompt: for parents of children with cancer? For health care providers? For other family members?
Ethicality: the extent to which the intervention has a good fit with an individual’s value system			Do you think there are any moral or ethical issues (moral or ethical consequences) related to offering the PairEd-Cb intervention? Prompt: in addressing parents from different sociodemographic backgrounds? Considering inequity in delivering the intervention? How do you evaluate cultural appropriateness, including the language of the intervention?
Perceived effectiveness: the extent to which the intervention is perceived to have achieved its intended purpose			Do you think the PairEd-C intervention will be effective for the family of children with cancer? What are the possible outcomes of the interventions? Prompt: Can we help get a better understanding of child illness, treatment, diagnostics, and treatment procedures? In coping with a child’s health condition? Improving overall parental and child health conditions? Improving parents’ capacity to provide care for their sick child?
Intervention coherence: the extent to which the participant understands the intervention and how it works			How do you predict the possible clarity (aim [purpose]) of the intervention for parents of children with cancer and/or for health care providers? How complex will the intervention be for parents of children with cancer and/or for health care providers?
**Opportunity cost: the benefits, profits, or values that would be given up engaging in the intervention**			
	Parents	What other priorities can the intervention possibly interfere with? Compared to different activities in the hospital, how much do you think this intervention needs priority? Parents priority? Health care providers’ priority? Was there anything that you/health care providers would possibly give up so that you receive the PairEd-C intervention? Prompts: For newly diagnosed? Child on follow-up? Inpatient vs outpatient?	
	For health care providers	What other priorities can the intervention possibly interfere with? Parents priority? Health care providers’ priority? Is there anything that you/parents of children with cancer would possibly give up so that you can provide the PairEd-C intervention? Prompts: For newly diagnosed? Child on follow-up? Inpatient vs outpatient?	
**Self-efficacy: the participant’s confidence that they can perform the behavior(s) required to participate in the intervention**			
	For parents	How confident are you that you will receive and complete the PairEd-C intervention? How confident are you using and understanding the education and teaching materials given by the PairEd-C intervention?	
	For health care providers	How confident are you delivering the complete PairEd-C intervention?	
Closing			Do you have any comments about the planned intervention? Is there anything that you think could be done better? Is there anything else you’d like to tell us? Any other additional concerns on its acceptability?

^a^OPD: outpatient department.

^b^Pair-Ed-C: Parent Education and Counseling.

### Qualitative Data Analysis

Qualitative data analysis will involve both deductive and inductive qualitative content analyses. Deductive thematic coding was used with a framework analysis technique based on the 7 constructs of the TFA [15]. In this phase, the text units were condensed, coded, and labeled using the participants’ own words as much as possible. Two independent researchers coded all transcripts, resolving discrepancies through consensus or discussion with a third party. During the coding process, quotes were determined to be generally positive, negative, or neutral toward the designed intervention. Similar codes were merged into key themes and categorized into domains of the TFA where applicable. Themes that did not fit within the constructs and domains of the TFA were also listed as new insights that emerged from the interview. In addition, continuous data analysis was performed following each in-depth interview. A saturation evaluation was conducted, and data collection ceased once data saturation was reached during analysis and no new categories were identified. Atlas.ti version 9 was used for data management. To ensure trustworthiness, we used triangulation, debriefing, and member checking [41]. Triangulation involved cross-verifying data from various sources, including parents, nurses, oncologists, and head nurses.

### Ethical Considerations

Ethical clearance was obtained from the Addis Ababa University College of Health Science institutional review board (protocol number 022/22/SPH). Permission was obtained from the TASH pediatric oncology unit. Written informed consent for the interviewee was obtained from each study participant. Participants were assured of their right to withdraw from the interview at any time, and participation in this study or refusal to participate did not affect their ability to access health services or any other services. Names and other personal information were not taken nor recorded. All information is kept confidential.

### Dissemination

This study’s findings will be published in an open-access journal and via national and international conference presentations.

## Results

This acceptability study is expected to show that the designed PairEd-C intervention will be acceptable for both HCPs and parents of children with cancer. In addition, we expect the findings can be used to improve the intervention before progressing to the next step of our project. The study was funded in 2017. We conducted the interviews with the study participants from December 2023 to January 2024. As of the acceptance of this protocol (June 2024), 12 participants, comprising 8 HCPs and 4 families of children with cancer, were interviewed. The manuscript will be completed and submitted for publication in early 2025.

## Discussion

### Overview

This study protocol describes the evaluation of the prospective acceptability of a newly designed intervention to improve FCC in the pediatric oncology setting in Ethiopia. A panel of experts in the field of pediatric oncology will modify the designed intervention based on information obtained from baseline surveys, international experiences, and their expertise. Although FCC is highly flexible and can be applied in a multitude of health care settings [42], studies on the design and evaluation of an FCC intervention in low-income countries are scarce. An intervention protocol to implement FCC in pediatric oncology settings is lacking in Ethiopia. Acceptability studies for health care interventions are becoming more widely recognized as a necessary condition [21], and the TFA has been successfully used to explore acceptability in health promotion interventions [28]. Therefore, this prospective acceptability study will establish strong foundational evidence that will play a vital role in the success of the newly developed intervention. In addition, the acceptability study will help to identify factors that would facilitate the acceptability of interventions to improve the designed family-centered education and counseling programs. The findings will also determine the strengths and weaknesses of the proposed intervention. We will also benefit from aligning the intervention with existing child cancer treatment.

### Strengths and Limitations

The strength of this study is that we used a widely used framework as guidance. Having an intervention developed based on locally generated information and experts who understand the available setup will facilitate the acceptability of the intervention. Involving health service leaders, HCPs, social workers, and parents of children with cancer will help obtain more comprehensive information about the possible acceptability of the designed intervention. Regarding possible limitations of the study, parents’ feedback might be biased due to their anticipation of trying something new. Social desirability bias might be introduced because of the nature of the study’s interviews. In addition, the transferability of the findings might be limited due to the purposive nature of participant selection.

### Conclusion

The results of this acceptability study will indicate that the designed intervention will be well received and accepted. The feedback obtained from parents, HCPs, and policymakers will be positive for all domains of the TFA. The education and counseling methods designed in the proposed intervention will significantly improve parents’ understanding of their children’s illness and enhance their capacity to provide care. It will also help fulfill the information needs of parents of children with cancer. In addition, we expect study participants will respond, as the intervention will help reduce parental psychological distress that is caused by their child’s diagnosis of cancer. Components of the proposed intervention, such as its detailed nature, inclusion of HCPs, peer educator training, delivery of information using multiple methods, and integrated delivery of the intervention with regular care, will help make it more acceptable among parents and HCPs. The outcome of this study will also help identify possible challenges that might affect the implementation of the study. Furthermore, the findings will help identify potential areas of improvement.

## Abbreviations

BCW: behavior change wheel
FCC: family-centered care
HCP: health care provider
MRC: Medical Research Council
PairEd-C: Parent Education and Counseling
TASH: Tikur Anbessa Specialized Hospital
TFA: theoretical framework of acceptability
TIDieR: Template for Intervention Description and Replication

## Data Availability

Data sharing does not apply to this protocol, as no data sets have been collected nor analyzed.
